# Medical education, reflections and perspectives from South Africa: a review

**DOI:** 10.1186/s12909-025-06910-8

**Published:** 2025-03-12

**Authors:** Danica Sims, Zukiswa Zingela, Mantoa Mokhachane, Gerda Botha, Dini Mawela, Veena Singaram, Karin Baatjes, Lionel Green-Thompson, Kerrin Begg

**Affiliations:** 1 Department of Education, University of Oxford, Oxford, UK; 2 Biomedical Engineering and Healthcare Technology (BEAHT) Research Centre, Faculty of Health Sciences, University of Johannesburg, Johannesburg, South Africa; 3 Faculty of Health Sciences, Nelson Mandela University, Gqeberha, South Africa; 4 Unit of Undergraduate Medical Education Faculty of Health Sciences, University of Witwatersrand, Johannesburg, South Africa; 5 Practice of Medicine, School of Medicine, Sefako Makgatho Health Sciences University, GaRankuwa, South Africa; 6 School of Clinical Medicine, University of KwaZulu-Natal, Durban, South Africa; 7 Department Surgical Sciences, Faculty of Medicine and Health Sciences, Stellenbosch University, Stellenbosch, South Africa; 8 Faculty of Health Sciences, University of Cape Town, Cape Town, South Africa

**Keywords:** South Africa, Medical education, Africa, Low-and-middle income countries, Curriculum transformation, Diversity, Inclusivity, Equity, Faculty development, Scholarship

## Abstract

Medical Education (ME) in South Africa has a century long legacy which continues to make a significant impact globally through its graduates. The aim of this paper is to showcase the evolving landscape of ME in South Africa, whiles addressing the legacy of socio-economic and educational disparities influenced by its colonial and Apartheid history. The paper focuses on the effectiveness of recent reforms to create a more equitable and inclusive healthcare education system that can meet the diverse needs of the population and prepare healthcare professionals for challenges in varied and resource-constrained environments. This reflection therefore contributes to setting the scene for the formulation of strategic objects for the future.

Key areas explored include widening access and participation through student admission and selection processes designed to promote inclusivity and equity; the development of student and academic support programmes to meet the needs of a diverse student population; the implementation of integrated, outcomes-based curricula; and the decentralisation of clinical training to underserved and rural areas. These measures aim to align medical training with a primary healthcare approach and to foster socially accountable and contextually responsive practitioners equipped to address local health challenges. Indeed, transformative learning experiences are shaping a resilient, agile and competent healthcare workforce.

However, this paper additionally identifies persistent challenges, including disparities in resource allocation, gaps in leadership and governance, accreditation and the tension between addressing historical inequities and ensuring robust academic standards. Moreover, the public healthcare system, which serves as the primary training ground for medical students, struggles to balance a quadruple burden of disease and infrastructural deficiencies with education needs.

Opportunities for growth are noted in the increasing emphasis on research and scholarship in ME, supported by the creation of a dedicated journal and five departments for medical and health professions education out of the ten medical schools in the country, formalising faculty development and postgraduate qualifications. This highlights the need for expansion of similar approaches in the other medical schools to address the gaps in research and the dearth of skilled clinician-educators.

Despite these strides, within the complexity of ongoing challenges, a focus on sustaining and strengthening robust quality assurance, a focus on primary healthcare, and expanding training of students and clinician-educators remains central.

## Introduction

South Africa (SA), often called the “rainbow nation”, is a complex country. The national motto, “*/xarra //ke”*, written in the indigenous Khoisan language, means “diverse people unite” – yet this multiracial democracy is the most unequal society in the world (Table 1). SA may best be known for Nelson Mandela – and *Apartheid* – and it is within this legacy that medical education (ME) will be reviewed. This system of ME has, anecdotally, provided graduates who have made outstanding contributions in clinical academic practice globally [1]. This paper engages with this illustrious legacy recognising that there is a growing tension emerging in the renewed drive for global accreditation [2]. We will celebrate the South African ME system and its global reach while reflecting on how it may better respond to the needs of its communities. This represents part of the voice of South African ME.

**Table 1:** Comparing contexts in which medical education takes place^a^: Crude measures of educational, socio-economic, health realities [3], adapted from Sims and Cilliers [4]

Indicators	USA	Canada	UK	Nether-lands	Aus-tralia	South Africa
**Economic and educational indicators**						
PIRLS^b^ (Progress in International Reading Literacy Study): international achievement in reading (2016)	549	-	559	545	544	320
TIMSS^b^ (Trends in International Mathematics and Science Study): international achievement in mathematics and science (2019)	528	518	523	528	524	364
GINI co-efficient (2022)^c^	41.1	33.3	35.1	28.1	34.1	63.0
Per capita GDP (USD)	63.6	43.3	41.1	53.4	51.7	5.7
**Resourcing and staffing of healthcare**						
Per capita health expenditure (USD)	10 921.01	5048.31	4312.89	5335.30	5427.46	546.69
Density of medical doctors (per 10 000 population)	26.1	23.1	28.1	36.1	36.8	9.1
Density of nursing and midwifery personnel (per 10 000 population)	145.5	99.4	81.7	111.8	125.5	13.1
**Burden of disease**						
Maternal mortality ratio (per 100 000 live births)	19	10	7	5	6	119
Under-five mortality ratio (per 1000 live births)	7	5	4	4	4	34
Neonatal mortality rate (per 1000 live births)	4	3	3	2	2	11
New HIV infections (per 1000 uninfected population)	0	0	0	0	0.04	4.94
Tuberculosis incidence (per 100 000 population)	3	5.6	8	5.3	6.6	520
Life expectancy at birth (both sexes; years)	78.6	82.8	81.4	81.6	82.9	63.6

^a^The USA, UK, Canada, Australia and Netherlands are consistently found to be the top producers of ME research outputs; yet the educational, economic and health contexts vastly differ from that of SA [5–8]

^b^Both PRILS and TIMSS use a scale from 0–1000, with a score of 625 generally indicating advanced educational achievement or performance, 550 for high achievement, 475 for intermediate achievement and 400 for low achievement

^c^The Gini co-efficient, a measure of statistical dispersion, measures income or wealth inequality. Lower values represent more equal wealth; higher values as less

### Education in South Africa

On examination of global university rankings, the inequalities in SA are mirrored in the Higher Education (HE) sector (CHE, 2021). The five top-ranked universities in SA, and the whole of Africa, are the historically White and advantaged universities. This divided reality sparked the “Fees-Must-Fall” and “Rhodes-Must-Fall” student protests in 2015 with calls for a free and decolonised HE in SA, and a collective reckoning with our ongoing colonial legacy. Recently, this has led to critical commentaries and calls for the decolonisation of ME – worldwide [9–12].

### Health and healthcare in South Africa

A quadruple burden of disease pressures the healthcare system in SA: communicable diseases (HIV/AIDS, tuberculosis, diarrheal disease); chronic illness (ischemic heart disease, diabetes mellitus) and mental health; injury and violence; and maternal, neonatal and child health [13, 14]. Dysfunction is rooted in historical (Dutch and British colonialism, segregation and Apartheid) and current challenges: discrimination and stigma, gender-based/violence, poverty, maldistribution of resources, poor quality of care, poor record-keeping, shortages of medicine and equipment, medical negligence, governance failures, poor leadership and management, inadequate human resource capacity and unequal social service delivery (piped water, sanitation, housing). Furthermore, macro- and socio-economic challenges such as slow progress in restructuring the segregated healthcare system post-Apartheid and increases in migration and urbanisation add to this burden [13, 15–17].

There is a growing private healthcare system in SA, funded by medical schemes and subsidised by the government, but the lack of integration between public and private healthcare sectors further worsens inequity in healthcare [13–15]. Importantly, health professionals working within the well-resourced private sector, which benefits the wealthy minority, are trained in the under-resourced public sector, with the poor and vulnerable carrying the burden of training the health workforce. Social responsiveness and social accountability literature from SA highlights the need for a social contract between practitioners and all of society [18].

The government has proposed a radical system of national health insurance (NHI) (in 2011) as a financing mechanism for the delivery of universal healthcare coverage to address the inequities and inefficiencies of the current system [14]. The proposed shift is rooted in the values of human rights, equity and social justice. It seeks to provide quality, responsive care using Primary Health Care (PHC), patient-centred, community-based, participatory, and public health approaches [19]. However, NHI implementation has been rocky due to gaps in ethical leadership, management and governance; poor quality of care and service delivery; malpractice and litigation threats; critical lack of human resources, healthcare financing and medical technologies; a scarcity of health information systems data and research, monitoring and evaluation, and quality improvement practices; and overall fragmentation leading to further delay [19, 20]. Despite slow improvements over time (e.g., under-5 mortality and life expectancy), SA remains committed to achieving all health-related Sustainable Development Goals [21].

### Medical education in South Africa

**Fig. 1:**
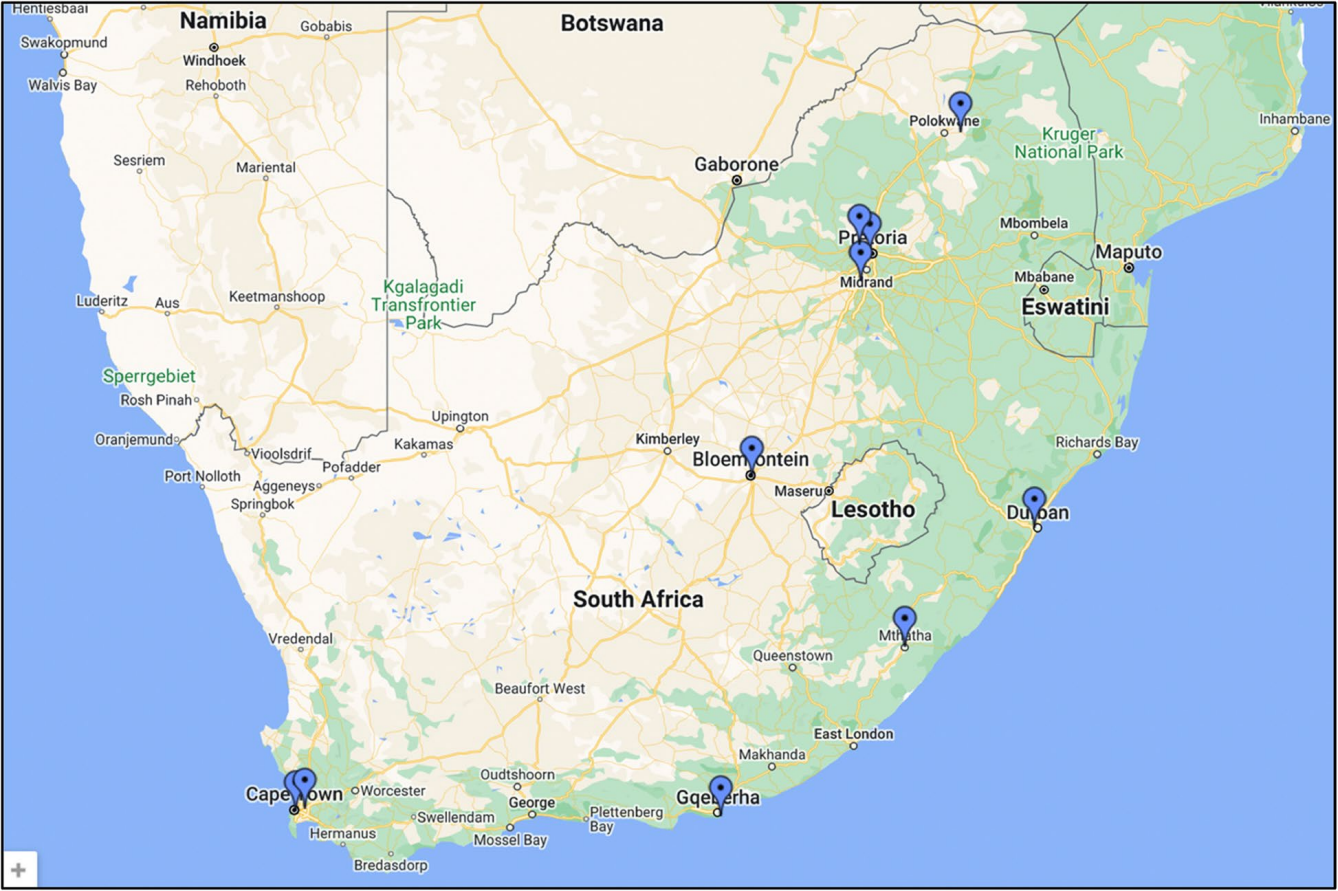
Geographical locations of medical schools in South Africa. There are ten medical schools in SA: two in the Western Cape, at Stellenbosch University (SU, established in 1955) and the University of Cape Town (UCT, 1912); two in the Eastern Cape, at Nelson Mandela Metropolitan University (NMMU, 2021) and Walter Sisulu University (WSU, 1976); one in Kwa Zulu Natal, at the University of Kwa Zulu Natal (UKZN, 1950); one in the Free State, at the University of the Free State (UFS, 1969); three in Gauteng, at Sefako Makgatho Health Sciences University (SMU, 2014), the University of Pretoria (UP, 1943) and the University of the Witwatersrand (WITS, 1919); and one in Limpopo, at the University of Limpopo (UL, 2005). Three provinces do not have medical schools. (Taken from https://www.gfmer.ch/Medical_search/Countries/SouthAfrica.htm; accessed 9 May 2023)

There are currently ten medical schools located in 6 (of 9) provinces in SA (see Fig. 1), offering a Bachelor of Medicine and a Bachelor of Surgery (MBChB or MBBCh). The oldest is 113 years (University of Cape Town), and the most recent opened in 2021 (Nelson Mandela Metropolitan University).

### Undergraduate medical education

#### Selection and admission

Students may apply for HE admission upon completing basic education. For medical programmes, selection evaluates specific subject achievements in applicants’ National Senior Certificates, commonly requiring: English (home or first additional language), mathematics, physical sciences (i.e., physics and chemistry) and usually natural sciences (i.e., biology). Many institutions also use the National Benchmark Tests (NBTs). NBTs benchmark students’ levels of academic literacy, quantitative literacy and mathematics according to the demands of HE standards. NBT performance has shown predictive validity for academic success in first-year medical students [22] and programme completion [23]. All institutions aim to make provision for disadvantage using forms of contextual data and weighting factors [24], including historical disadvantage based on demography (e.g., race and rural origin), educational disadvantage (e.g., school quintiles and fee status, with quintiles 1, 2 and 3 and no-fee paying schools as indicators of community poverty, low resourced schools and poorer basic education quality) and economic disadvantage (e.g., households receiving social or disability grants as an indicator of low household income).

In the pursuit of redress of previous disadvantage, racial transformation, inclusivity and equity remain central to the selection of students [24]. This aspires to deliver a more representative graduate population and the preparation of graduates for service in underserved communities [25]. In 2015, the racial profile of medical students in SA was reported as 39% Black, 33% White, 13% Coloured (a non-offensive term for mixed race heritage) and 13% Indian/Asian, with the majority female (62%) [24]. Critically, the number of Black students in undergraduate ME in 2015 was still significantly lower than the general population and other fields of HE (e.g., in 2024 Black South Africans account for 81.4% of the general population; and by 2020 Black students enrolled in HE institutions in SA represented 79.4% of the total number of students) [24, 26, 27]. Additional research on enrollment and throughput statistics in ME are needed to allow year-on-year analyses and thorough reflections about the reasons why this discrepancy may still exist.

Acceptance into medical programmes is competitive in SA due to the limited available capacity (1–1.5% acceptance rate), observed in the establishment of more medical schools in recent years. Intake numbers are regulated by the Health Professions Council of South Africa (HPCSA), with medical schools accepting anything from 50–350 students per year (newer medical schools accept lower numbers than more established programmes). While entry is competitive, institutions seek to intentionally widen access and participation through recruiting diverse candidates with open-days, local school visits and outreaches. Furthermore, some institutions implement affirmative action policies that incorporate quota systems for students from previously disadvantaged backgrounds and rural communities.

Tension remains in balancing equity and redress with challenges around retention and throughput of students with a poor-quality foundation of basic education. On the one hand “fixing the numbers” may have resulted in more medical schools enrolling more Black students, however this expansion has not necessarily resulted in a more equitable system in terms of outcomes (e.g., number of Black students graduating). This expansion needs to be accompanied with concomitant investment in the effectiveness of teaching and learning, student support, as well as strategies and interventions for reducing drop-out rates and improving throughput and graduation rates [28]. Both the National Student Financial Aid Scheme (NSFAS) and enrolment into foundation and extended curricula programmes (ECPs) speak to address this (see Programme structure and duration section) [29]. Furthermore, academic support and peer-mentoring programs, which pair senior students with new or junior students, aim to provide both academic and psychosocial support.

NSFAS was first introduced in 1991 and expanded substantially in 2015 after the “Fees-Must-Fall” campaigns to make HE more affordable for poor students in SA. NSFAS substantially contributed to increasing access and supporting the retention of poorer students in the system until they obtained their qualifications [27]. ECPs prioritise the admission of students with high potential who may have underachieved due to poor schooling [30].

ECPs are formal undergraduate degree programmes where the minimum duration is lengthened by 6–12 months to provide substantial academic work of a foundational and developmental nature in addition to the coursework prescribed for the regular curriculum. This is designed to strengthen the academic foundations needed for underprepared students to have a greater chance of succeeding in undergraduate training. Whether medical schools offer ECPs or not, it is important that all continue to provide appropriate and timely interventions for entrants from diverse educational backgrounds. It is furthermore important to note that this paper does not include a reflection on the Capabilities Approach (CA), which may add to the debate around selection and admissions. CA, as normative framework with a social justice orientation, allows consideration of individual well-being as well as social institutions, policies, and contexts that may influence individual student’s accessing HE institutions (e.g. who decides, which criteria are being used, what influences a student’s decision on where to apply to, etc.) [31]. This therefore indicates a need for further research.

#### Programme structure and duration

**Fig. 2:**
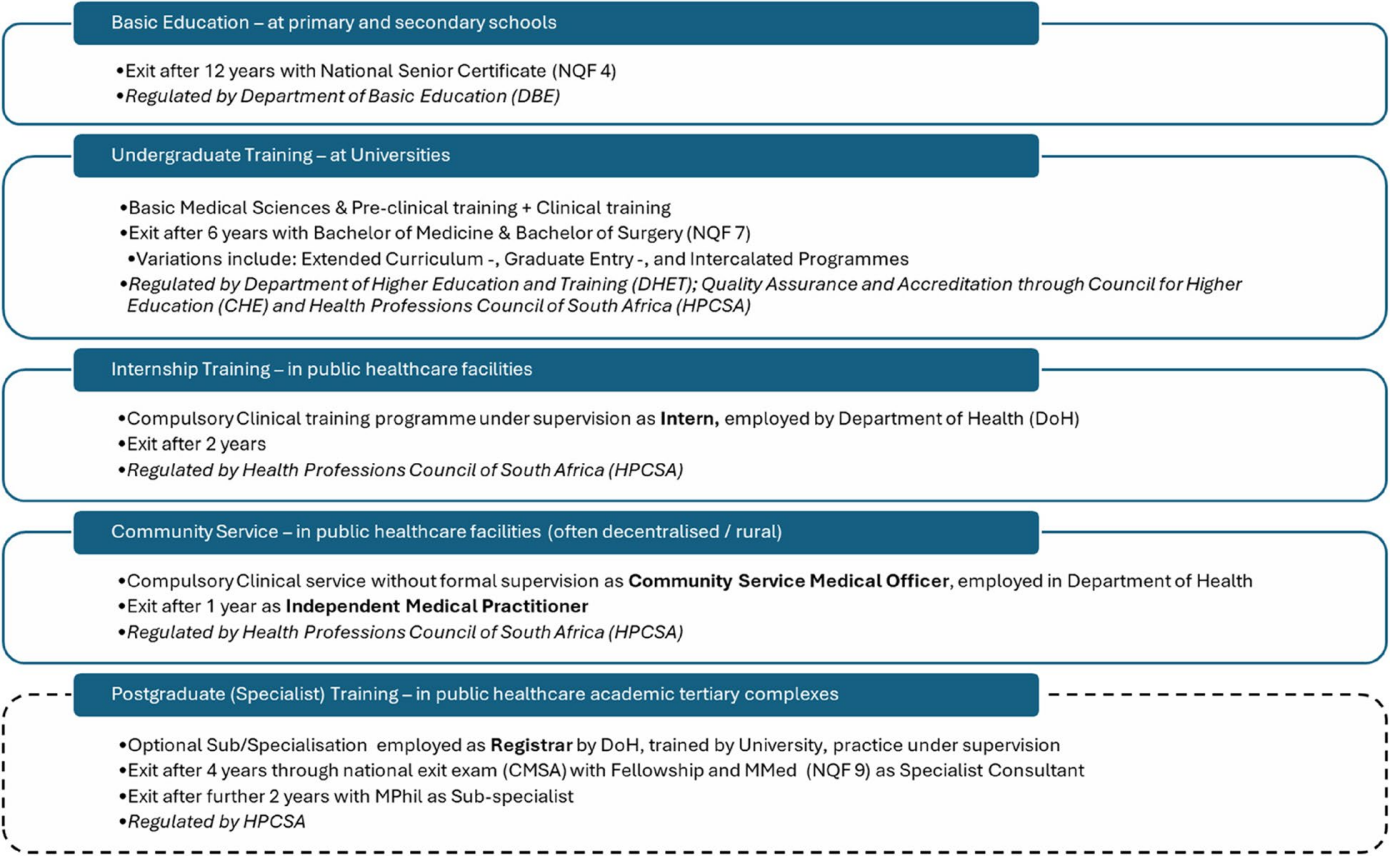
The structure, timing and regulation of ME in SA. Students can enter medical training directly after graduating from high school or with a previous degree. In general, undergraduate medical degrees span six (6) years of training (exceptions: a 5-year programme at UFS and WITS graduate entry medical programme of 4 years), followed by an additional mandatory three (3) years of public health system training: two (2) years of clinical internship and one (1) year of community service. Only once these compulsory nine (9) years of training are completed are graduates certified to practice independently as medical practitioners. Further formal postgraduate specialist and sub-specialist training is available (but not compulsory) and is beyond the scope of this article. Throughout undergraduate training, trainees are referred to as “medical students”, whereas, upon graduation and entry into public clinical training and service they are called “interns”, followed by “medical practitioners”. During postgraduate training, doctors are referred to as “registrars” until they complete their specialist qualification and then are called “consultants”. NQF: National Qualifications Framework; DBE: Department of Basic Education; DHET: Department of Higher Education and Training; HPCSA: Health Professions Council of South Africa; DoH: Department of Health; CMSA: Colleges of Medicine of South Africa

**Fig. 3:**
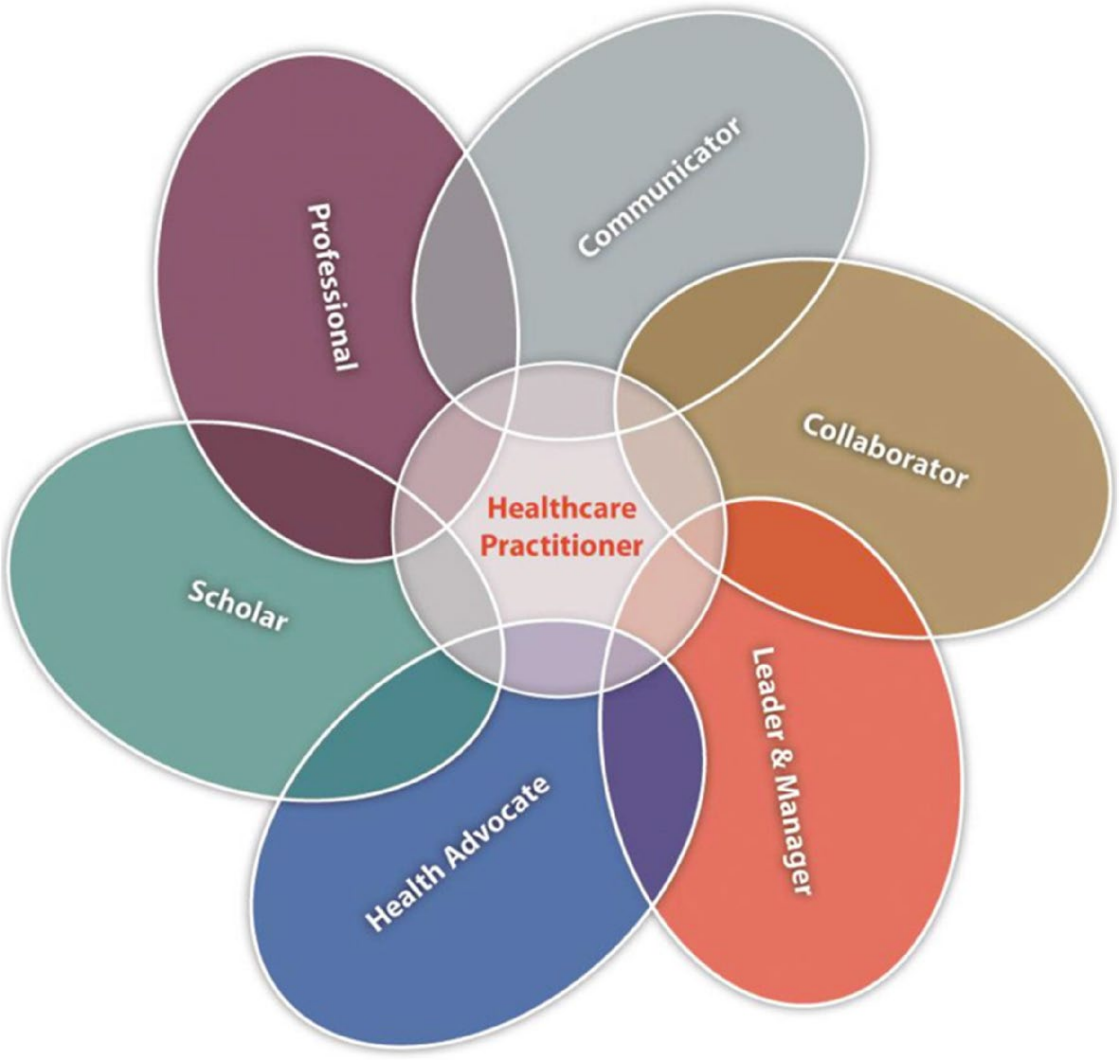
Afri-MEDs competency framework [54]

Mandatory accreditation by HPCSA (an accreditation agency with a well-established history of service) and Council for Higher Education (CHE) (accredits HE since the 1994 democratic transition) serves as a quality assurance mechanism promoting professional and public confidence in the quality of ME, it assists medical schools in attaining desired standards, and it ensures that graduates′ performance complies with national norms [32]. The HPCSA prescribes core competencies that should be incorporated in the training of undergraduate medical students in SA (Fig. 3), however it does not prescribe the structure nor the duration of the training programme to be followed by medical schools. To practice as a medical doctor, nine years of compulsory training are generally required: six of undergraduate medical training, followed by three years of public healthcare service and training (Fig. 2). The exception is a five-year programme at UFS (see Table 2). There are additional programme variations, including extended curricula, graduate entry and intercalated programmes. Requiring medical graduates to practice in the public healthcare system (a mandatory two years for internship and one year for community service) before receiving full medical licensure, reflects broader trends in Africa [33, 34].

**Table 2:**
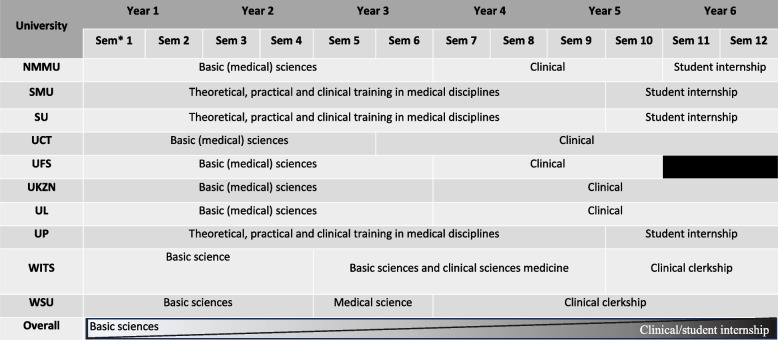
Undergraduate medical degree programme structures at South African medical schools

^*^Sem: semester

As aforementioned, most medical schools have ECPs (also termed intervention, development or bridging programmes) for students who come from disadvantaged schools or fail the first semester/year of the standard programme [25, 35, 36]. This adds another year to their training to build a solid academic foundation for successful student progression through the remaining programme [37].

WITS offers a four-year graduate entry medical programme to students who already have a Bachelor’ degree in the field of Science with these students joining the third year of the MBBCh programme. WITS recently introduced an intercalated PhD in the last two years allowing students to graduate as clinician scientists. While no graduate entry programme exists at UCT, graduates are sometimes accepted into the second year of the standard MBChB programme [24], and they offer an intercalated Bachelor of Science in Medicine Honors programme between the third and fourth years of the standard MBChB programme [38] to support the training of clinician-scientists, which can also be vertically articulated into Master and PhD programmes.

#### Curriculum

Medical programmes consist of an integrated systems-based curriculum [36]. The first one to three years focus more on pre-clinical basic medical sciences. Some programmes include courses with a social sciences focus, such as indigenous languages (SA has eleven official languages), professionalism, interprofessional teamwork (i.e., joint training with allied health professions), and medical health humanities [39, 40]. The second component of the programme incorporates a greater focus, time and effect on clinical rotations (or clerkships) of variable lengths. Additionally, some medical schools also offer elective courses in which students may choose or organise an elective of their choice (clinical, research or public health) locally or internationally. While there is structure variation between medical schools, the principle remains similar: spiraling from basic medical sciences during years 1 and 2 to theoretical and practical clinical training in years 3 and 4 to clinical consolidation in student internship (“clerkships”) during years 5 and 6 on the clinical platform (e.g., primary, secondary and tertiary level public health/government clinics and hospitals). Therefore, the ‘divide’ between basic sciences and clinical medicine are somewhat artificial, as a continuum across the years and semesters showcase the spiral curriculum in alignment with modern medical curricula described in international literature [41] (Table 2 “overall”). While some authors emphasised that the amount of time spent in real-life clinical settings needs to be increased and needs to start earlier in the curriculum [42], others found clinical exposure through early and longitudinal clinical contact in a range of public health care systems (i.e., primary to tertiary care levels) including in resource-constrained communities (e.g., informal settlements, rural and remote communities) already exists as mechanism towards social responsiveness [43].

Despite having integrated and spiraled curricula, reviews still report fragmentation and a lack of continuity in developing core competencies, for instance, evidence-based healthcare knowledge and skills [44], health systems understanding [45] and inconsistent alignment with primary healthcare in undergraduate community-based training [46]. However, others have reported curriculum changes and improvements in light of previous reviews, closing the clinical skills theory–practice gap [42].

The HPCSA prescribes six competencies for all healthcare practitioners called ‘Afri-MEDs’: communicator, collaborator, leader and manager, professional, researcher and scholar, and health advocate (Fig. 3). Wary of directly applying competency frameworks developed in the global North in a vastly different context, Afri-MEDs was adapted from the Canadian Medical Education Directive for Specialists (CanMEDS) to suit the South African context – with debates around its appropriateness [47]. For example, for communication, while all programmes are taught in English, courses in Indigenous languages are elements of undergraduate curricula (e.g., Afrikaans, isiXhosa, isiZulu, Setswana) [48, 49] – yet, indigenous language proficiency and intercultural communication remain areas of curricular improvement [50]. Similarly, professionalism and professional identity are being redefined to align with the South African context and broader Indigenous African philosophy [51–53].

#### Teaching and learning

Considering the broader context of SA, it is not surprising that an equity-driven Primary Health Care (PHC) and biopsychosocial approach have formed the foundation of SA’s national health policy since 1994’s democracy and thus been integrated into medical curricula across the country (e.g., community-based, public health and PHC foci of preventative, promotive, curative and rehabilitative healthcare) [25, 36, 55]. Other approaches and pedagogies used are outcomes-based education, case- and problem-based learning, student-centred and self-directed learning, peer-assisted learning, small group learning, multi-disciplinary and inter-professional education, and patient-centred education [36, 56].

Clinical training takes place on clinical platforms within the public healthcare system at Primary (community health clinics), Secondary (district and regional hospitals) and Tertiary (central hospitals) healthcare facilities. It includes bedside teaching, ward rounds, ambulatory care practice learning, tutorials, simulations, etc. [43].

The teaching platform has expanded to include decentralised clinical training, where students take clinical rotations or longitudinal clerkships in underserved rural and peri-urban settings to address health system needs [57]. Medical schools in SA utilise various decentralised platforms for undergraduate training, but there are differences in the type and nature of investments in these platforms. Most training is hospital-based, with utilisation of associated PHC facilities constrained by insufficient resources, and competition between disciplines (e.g., medical students versus nursing, physiotherapy, occupational therapy, speech and language therapy, audiology, etc. students). Decentralised training requires an explicit vision, and institutional investments in human resources, finances, and infrastructure [58]. This has shifted training towards population-based and interprofessional education approaches; providing more authentic workplace-based learning for students immersed in a local context as an integral part of the multi-disciplinary healthcare team with opportunities for more hands-on and collaborative learning experiences [58–62]. This aligns with the broader mission to “transform” ME in SA using PHC, community-based and socially accountable educational approaches [18, 36, 59, 63–65]. Rural placement of students presents access to healthcare for rural patients, and as a collaborative training approach, assists students to respond in future as socially responsive citizens [66]. Decentralised training not only decreases the urban–rural workforce maldistribution by increasing capacity in under-resourced and overburdened public health system settings [29], but equips graduates as fit-for-purpose [57], retaining their service in rural and underserved settings [58]. However, short-term, silo-placements during clinical years may insufficiently prepare students to address systemic quality of care and health disparities. Medical schools should therefore ensure relevant exposure through longer, integrated placements with a multi-disciplinary approach; and teach students skills towards collaboration, leadership, and management through community-engaged partnerships [43].

#### Assessment

Assessment approaches include formative, continuous assessment practices (theoretical and practical tests, tutorials, laboratory reports, reflective journaling, projects, seminars, logbooks, case studies, patient write-ups, portfolios, clinical attendance, mini-CEXs, DOPS) over a block of time (e.g., clerkship, semester, year) with final, summative written assessments (including MCQs, SAQs, EMIs, essays) and clinical examinations (including OSPEs, OSCEs, orals, long cases) at the end of that time period. One medical school reported MCQ (76.9%), OCSE (53.6%) and workplace-based assessment (WBA) (30.1%) as the most widely used assessment methods, with feedback and post-assessment moderation practices remaining poor [67].

Despite the range of assessment practices employed, South African researchers have recommended cost-effective adaptations (e.g., orals, portfolios and WBA with real and not standardised and simulated patients, over OSCEs for instance) [68–71]. Therefore, fit-for-purpose (i.e., high-fidelity workplace-based clinical assessments) and fit-for-context assessments [71, 72] (i.e., culturally-appropriate, locally responsive) remain an aspiration in our resource-constrained environments [25, 68, 69].

#### Curriculum review

Both the HPCSA and the Council Higher Education prescribe ongoing curriculum self-review and quality assurance of training in SA with specifications for frequency, review teams, process and templates to be used. Several programme and curriculum revisions have taken place, with core content evaluated for contextual relevance for safe, effective, and evidence-based practices [25, 73]. It was found that concepts and competencies related to public health, health systems research, evidence-based healthcare, and infection prevention and control are important to produce graduates who can act as change agents within the health system. However, most of these health systems strengthening competencies as well as health leadership, management and health advocacy were not taught sufficiently or introduced late and, in addition, teaching was fragmented, with a lack of continuity and progression of learning across the curriculum. There are limited opportunities for experiential learning, and little integration into clinical teaching [43, 74]. Additionally, beyond clinical competency, personal and social competencies should be more strongly embedded in curricula to prepare graduates to transition from undergraduate training to clinical internship [75]. Some medical schools have also embarked on curriculum mapping processes and these can potentially assist in drawing data for curriculum review and possible benchmarking once complete [76].

#### Adapting and adjusting to disruptions to education

Briefly, with regards to COVID-19 and emergency remote teaching (ERT), student access and online readiness, within our context of inequity, was of concern [77]. Implementing ERT, especially regarding assessment and clinical training, was challenging and exacerbated inequity of the most disadvantaged and at-risk students [78, 79]. However, perceptions and experiences of both staff and students of online ME have been largely positive [80, 81] with recommendations for blended approaches for effective bridging of the theory–practice gap, including clinical skill domains [82]. Regarding the emergence of artificial intelligence (AI) in ME, and other digital technology innovations like extended reality (XR), while there is potential to diversify and personalise ME, and enhance healthcare delivery within SA [83], a lack of related policy, along with limited resourcing, capacity and equity concerns, remain significant barriers to adoption [84].

### Accreditation and certification

Endorsement and accreditation certification of ME in SA id done through complementary educational (general) regulatory bodies and professional regulation.

#### Educational accreditation

The Council for Higher Education (CHE) is an independent statutory body established by the Higher Education Act, No. 101 of 1997, as amended. It is also governed by the National Qualifications Framework (NQF) Act No. 67 of 2008 which declared it the Quality Council for Higher Education. The statutory quality assurance responsibility for the accreditation of the programmes of HE institutions, is assigned to the Higher Education Quality Committee (HEQC) by the Higher Education Act of 1997 as well as by the Education and Training Quality Assurer (ETQA) and regulations of the South African Qualifications Authority (SAQA). Programme accreditation entails the evaluation of HE academic programmes in accordance with the HEQC’s programme accreditation criteria, which stipulate the minimum requirements for programme input, process, output and impact, and review.

The NQF is the system that records the credits assigned to each level of learning achievement in a formal way. The NQF is designed to help students move more easily between programmes as they pursue their education and to address past discrimination in education, training, and employment. The Higher Education Qualifications Framework (HEQF) and the Higher Education Qualifications Sub-Framework (HEQSF) are part of the NQF and aim to determine the qualification types, characteristics and purposes of all HE qualifications in SA.

Institutional audit, on the other hand, which is also within the HEQC’s jurisdiction, evaluates an institution’s policies, systems, strategies and resources for quality management of the core functions of teaching and learning, research and community engagement, using the HEQC’s audit criteria. Quality management encompasses arrangements for quality assurance, quality support and enhancement, and quality monitoring, and covers aspects of input and process as well as outcomes.

Clearance for, quality assurance and accreditation of all HE training programmes in SA is therefore the responsibility of various governing authorities. The role of the Department of Higher Education and Training (DHET) should also be clarified as it provides clearance for programmes to be developed once HE institutions identified the need and provided a rationale for such. All HE qualifications at all levels (e.g. higher certificate, diploma, bachelors, post graduate diploma, masters and doctorate degrees), should be registered with DHET and accredited by CHE, HEQC and SAQA. All undergraduate bachelor medical training programmes in SA currently carries full accreditation status as can be observed on the SAQA website https://regqs.saqa.org.za/.

Understanding of the role and functions of the various HE statutory bodies, and who to approach for what, may seem overwhelming for the individual lecturer or medical expert that see a need for training of a specific cadre of medical professionals. As such, most institutions have established units with expertise to guide the medical fraternity in programme development, review and accreditation. Even so, the completion of all templates by the medical experts are seen as cumbersome and add to their already overburdened workload. One concern may be the lack of SAQA accreditation of all sub-specialist training programmes (these are highly specialised and focus on medical specialists who already have a Masters Degree in Medicine) and therefore further discussion about this concern falls outside the scope of this manuscript but is mentioned as it will need action by medical schools and the relevant Colleges of Medicine in South Africa (CMSA).

It must also be mentioned that there are tensions between DHET and NQF system, which are often at odds with professional education.

##### Professional regulation

The HPCSA is a statutory body that oversees the education, training and registration of all practicing health professions in SA, in line with the Health Professions Act 56 of 1974. There are twelve professional boards within the HPCSA body, with the Medical and Dental Board having oversight of medical practitioners.

Registration with the HPCSA is a prerequisite for professional training and practice at all stages: student, student-internship (i.e., training on the clinical platform or a clinical elective), internship, community service, independent practice, supervised specialist-in-training (i.e., registrar), and specialised or sub-specialist practice (i.e., consultant). The HPCSA should furthermore endorse all new qualifications that are being developed by medical schools for its submission to DHET (for programme qualification mix clearance) and to CHE/HEQC and SAQA for accreditation. The HPCSA also oversees registration of clinical training sites and the number of trainees for all levels of qualifications, training platforms and sites. Medical schools are expected to submit annual reports based on previous HPCSA certification of training and regular self-reviews are required. Accreditation procedures as set out by the Liaison Committee on Medical Education in the USA by 2006 were similar to those set in SA, as well as to other countries in Sub-Saharan Africa, however the focus was more on process of accreditation than on outcomes of training [32].

Both educational and professional registration and accreditation processes should be flexible and require ongoing faculty development about the intention and processes involved, as well as financial support so that accreditation of medical programmes in our context can be responsive to the needs of our patient populations. Accreditation standards therefore should increasingly include a focus on social accountability.

Furthermore, regional cooperation and international accreditation agencies seem to be a growing trend worldwide. Transnational initiatives support physician migration, mutual degree recognition, and the sharing of academic resources as technology becomes increasingly accessible. For instance, the World Federation of Medical Education (WFME) provides recognition to accreditation agencies [85]. However, we, and others, critique this global accreditation movement, for it can perpetuate a colonial discourse of ‘West is best’ and further falsely paint medical education and training in the global South as deficient and sub-parr [2, 86]. Aligned to longstanding transformational HE agendas, and more recent calls to decolonise the field, we and others advocate for prioritising national needs over international pressures [87, 88] – and indeed, drivers for the evolution of ME in SA have largely been due to internal factors and not external influences. Throughout this paper we have evidenced robust, nuanced and contextually responsive undergraduate ME as an example of quality training outside of the global North. Indeed, many countries from the global North find South African trained medical graduates to be competent, agile and resilient – seen in the ‘poaching’ of qualified doctors (with negative financial and health implications for SA) – which, while an implicit recognition of the quality of our training, they remain silent on explicit recognition of our educational systems.

### Medical education research (MER)

Challenges around MER in South (and sub-Saharan) Africa include the sustainable creation and maintenance of such units; expanding and building capacity of the limited qualified workforce; a dependence on external funding and a lack of local government institutional financial support [29, 89]; a need for strong leadership [29, 46, 89, 90]; a lack of recognition and reward in comparison to disciplinary research activities [36]; and the pressure to conform to global trends as opposed to undertaking locally-relevant and responsive MER [87, 91].

Despite these challenges, MER is growing in SA with five medical schools (SU, UCT, UFS, WITS, UKZN) having departments, centres or units of Health Sciences Education (HSE), Health Professions Education (HPE) or ME. Reflective of contextual needs and resource constraints, these MER department serve multiple functions: support (i.e., faculty development), leadership (i.e., curriculum reform), teaching and supervision (i.e., postgraduate qualifications), and research. This is demonstrated in regional MER priorities: provision of training that addresses health and health system needs; interprofessional education; holistic person-centred approaches to healthcare; use of technology in ME; clinician-educator faculty development; quality assurance; resourcing for MER; curricula responsiveness; rural training; and the relevance of communication training in culturally-diverse settings [92]. Yet, to meet these priorities, first an enabling national environment for undertaking research must be created, including providing sufficient research resourcing and training [92].

While MER is growing in SA, evidenced by creation of a dedicated journal, the African Journal of Health Professions Education (AJHPE), in 2014, and increasing outputs [8]; in comparing SA with global trends, there is still an under-representation of ME publications from researchers in sub-Saharan Africa [5, 7, 93]. In turning towards the international environment of MER, Southern scholars face challenges such as exclusion when seeking to disseminate their research [94, 95], in which has been recognised by recent calls for improving justice, equity, diversity and inclusion in global scholarship [96–99]. As with our critique of global accreditation movements, so we bring decolonial and transformational (i.e., reparative) recommendations to the fore: we cannot publish in international journals in the global North, with a lack of diverse global South representation on editorial boards [98, 100, 101], if there is a continued devaluation and delegitimisation of our praxis. This submission reflects this reality: this specific paper is possible because of this journal’s recognition of exclusion and a lack of representation of scholarship from low-and-middle income countries – yet the category of a ‘special edition’ underscores the divide and two-tier system. Similarly, despite calls for researchers to cite scholarship from the global South [97], how can we be cited if our submissions are rejected or, if accepted, we cannot afford to pay (even the subsidised) open access publication fee? Unless the global community takes collective responsibility, with material consequences, for equitising the field, growing MER from the global South and low-and-middle income countries will remain an uphill battle.

## Conclusion

There are persistent challenges in SA’s healthcare system which continue to impact on ME. These include: student selection, retention and throughput [102]; developing and retaining contextually-responsive, resilient, empathetic, socially-accountable health professionals [103]; tensions between equity and redress and the training of students in difficult healthcare systems and the ethical dilemmas they experience. Other challenges relate to the demand for an increasing health workforce to meet the growing population, burden and complexity of disease, especially in the public sector; addressing the urban–rural maldistribution of healthcare workers to equitably meet the needs of underserved communities; improving interprofessional education and collaborative practice; incorporation of the internship and community service years as postgraduate study within HEIs and opportunities to differentiate into specialist training earlier on; faculty development in this mission [29]; and, improving human resources, leadership, governance, infrastructure and financing of HPE for health systems strengthening [29, 57, 58, 90].

We are proud to report that these recommendations are already prioritised and embedded within medical education and training in SA. In the short term, to enhance equity, retention, and throughput for disadvantaged and underrepresented students, we recommend reinforcing student support systems. This includes academic support through ECPs, social support via peer mentoring, and financial assistance through NSFAS. To improve healthcare system resourcing, rural service delivery, and contextually responsive competency development, it is essential to expand integrated, interprofessional, longitudinal, and decentralised clinical training, accompanied by regular curriculum reviews. Furthermore, to address the increasing demand for more doctors in training and enhanced scholarship, faculty development for clinician-educators must be improved. This improvement should include funding for qualifications and incentives for participation, revisions to workload models, and enhancements in recognition and career advancement opportunities. These measures should be supported by increased transparency and efficiency in accreditation and governance processes. In the longer-term, systemic issues must be addressed, including establishing South-South partnerships for the development of regional accreditation standards, and the strengthening and amplification of our voices on a global stage [104] – diversifying discourses by bringing decolonial and Indigenous perspectives to the field.

In conclusion amidst these challenges, we celebrate ME in South Africa as an example of excellent internationally comparative education, with ongoing complex challenges, and our responses, as opportunities and an impetus for transformative learning and educational research that leads to the training of a more resilient, adaptable and stronger future healthcare workforce [105].

## Data Availability

No datasets were generated or analysed during the current study.

## References

[1] George A, Blaauw D, Thompson J, Green-Thompson L. Doctor retention and distribution in post-apartheid South Africa: tracking medical graduates (2007–2011) from one university. Hum Resour Health. 2019;17:1–9. 31842879 10.1186/s12960-019-0439-4 PMC6916458

[2] Rashid MA, Griffin A. Is West Really Best? The Discourse of Modernisation in Global Medical School Regulation Policy. Teach Learn Med. 2023;36(4):1–12. 10.1080/10401334.2023.2230586 37401838

[3] WHO. World Health Statistics 2020: Monitoring health for the Sustainable Development Goals. 2020.

[4] Sims D, Cilliers F. Clinician educators’ conceptions of assessment in medical education. Adv Health Sci Educ Theory Pract. 2023;28(4):1053–77. 36662334 10.1007/s10459-022-10197-5 PMC10624725

[5] Tutarel O. Geographical distribution of publications in the field of medical education. BMC Med Educ. 2002;2(3):3. 12031092 10.1186/1472-6920-2-3 PMC113258

[6] Rotgans JI. The themes, institutions, and people of medical education research 1988–2010: content analysis of abstracts from six journals. Adv Health Sci Educ Theory Pract. 2012;17(4):515–27. 21971993 10.1007/s10459-011-9328-x

[7] Doja A, Horsley T, Sampson M. Productivity in medical education research: an examination of countries of origin. BMC Med Educ. 2014;14:243. 25404502 10.1186/s12909-014-0243-8 PMC4239316

[8] Maggio LA, Costello JA, Ninkov A, Frank JR, Artino AR. The Voices of Medical Education Science: Describing the Published Landscape. Med Educ. 2022. 10.1111/medu.14959 PMC10098831 36282076

[9] Naidu T. Modern Medicine Is a Colonial Artifact: Introducing Decoloniality to Medical Education Research. Acad Med. 2021;96(11S):S9–12. 10.1097/ACM.0000000000004339 34380933

[10] Paton M, Naidu T, Wyatt TR, Oni O, Lorello GR, Najeeb U, et al. Dismantling the master’s house: new ways of knowing for equity and social justice in health professions education. Adv Health Sci Educ Theory Pract. 2020;25(5):1107–26. 33136279 10.1007/s10459-020-10006-x PMC7605342

[11] Naidu T. Says who? Northern ventriloquism, or epistemic disobedience in global health scholarship. Lancet Glob Health. 2021;9(9):e1332–5. 34416216 10.1016/S2214-109X(21)00198-4

[12] Naidu T. Southern exposure: levelling the Northern tilt in global medical and medical humanities education. Adv Health Sci Educ Theory Pract. 2021;26(2):739–52. 32500281 10.1007/s10459-020-09976-9

[13] Coovadia H, Jewkes R, Barron P, Sanders D, McIntyre D. The health and health system of South Africa: historical roots of current public health challenges. Lancet. 2009;374(9692):817–34. 19709728 10.1016/S0140-6736(09)60951-X

[14] Mayosi BM, Lawn JE, van Niekerk A, Bradshaw D, Abdool Karim SS, Coovadia HM, et al. Health in South Africa: changes and challenges since 2009. Lancet. 2012;380(9858):2029–43. 23201214 10.1016/S0140-6736(12)61814-5

[15] Maphumulo WT, Bhengu BR. Challenges of quality improvement in the healthcare of South Africa post-apartheid: A critical review. Curationis. 2019;42(1):e1–9. 10.4102/curationis.v42i1.1901 PMC6556866 31170800

[16] Lebese L, Begg K, Dudley L, Mamdoo P, Engelbrecht J, Andrews G. Development of a national strategic framework for a high-quality health system in South Africa. South African Health Review. 2018;2018(1):77–85.

[17] Begg K, Andrewsi G, Mamdoo P, Engelbrecht J, Dudley L, Lebesei L. Development of a national strategic framework for a high-quality health system in South Africa. Durban: Health Systems Trust; 2018. p. 77–86.

[18] Green-Thompson LP, McInerney P, Woollard B. The social accountability of doctors: a relationship based framework for understanding emergent community concepts of caring. BMC Health Serv Res. 2017;17(1):269. 28403860 10.1186/s12913-017-2239-7 PMC5389126

[19] Shisana O, Dhai A, Rensburg R, Wolvaardt G, Dudley L, Patel RH, Grobler GP, Masilela TC, Rispel LC, Puren A, Stewart J, English R, Whittaker S. Achieving high-quality and accountable universal health coverage in South Africa: a synopsis of the Lancet National Commission Report. S Afr Health Rev. 2019;2019(1):69–80. 10.10520/EJC-1d2ab57a7e.

[20] Passchier RV. Exploring the barriers to implementing National Health Insurance in South Africa: The people’s perspective. S Afr Med J. 2017;107(10):836–8. 29022524 10.7196/SAMJ.2017.v107i10.12726

[21] Achoki T, Sartorius B, Watkins D, Glenn SD, Kengne AP, Oni T, et al. Health trends, inequalities and opportunities in South Africa’s provinces, 1990–2019: findings from the Global Burden of Disease 2019 Study. J Epidemiol Community Health. 2022;76(5):471–81. 35046100 10.1136/jech-2021-217480 PMC8995905

[22] Mabizela SE, George AZ. Predictive validity of the National Benchmark Test and National Senior Certificate for the academic success of first-year medical students at one South African university. BMC Med Educ. 2020;20(1):152. 32404200 10.1186/s12909-020-02059-8 PMC7218523

[23] Mabizela SE. Green-Thompson L. Exploring the association of the National Benchmark Test results with the academic performance of medical students who completed the degree in minimum time Journal of Education. 2019;75:44–55.

[24] van der Merwe LJ, van Zyl GJ, St Clair Gibson A, Viljoen M, Iputo JE, Mammen M, et al. South African medical schools: Current state of selection criteria and medical students' demographic profile. S Afr Med J. 2015;106(1):76–81. 10.7196/SAMJ.2016.v106i1.9913 26792312

[25] Hartman N, Kathard H, Perez G, Reid S, Irlam J, Gunston G, et al. Health Sciences undergraduate education at UCT: a story of transformation. S Afr Med J. 2012;102(6):477–80. 22668942 10.7196/samj.5680

[26] sa s. Statistical release P03014 Census 2022. Republic of South Africa: Department of Statistics South Africa; 2023.

[27] de Villiers P. Perspective chapter: The role NSFAS played to facilitate poor students in South Africa. In: Waller L, Waller S, editors. Higher Education - Reflections from the Field. 2: IntechOpen; 2023. p. 1–24.

[28] MacGregor RG, Ross AJ. Throughput rates and time to completion of health science students of rural origin in South Africa. Afr J Health Prof Educ. 2024;16(2):46–53.

[29] Volmink J. Reconceptualising health professions education in South Africa. S Afr J Sci. 2018;114(7–8):4–5.

[30] Ogude NA, Rollnick M. Ideological positioning of Extended Curriculum Programmes – a case study of a large South African research university. South African Journal of Higher Education. 2022;36(2):222–38.

[31] Mahlangu V. Rethinking student admission and access in higher education through the lens of capabilities approach International Journal of Educational Management. 2019;34(1):175–85.

[32] Cueto J, Burch V, Adnan N, Afolabi B, Ismail Z, Jafri W, et al. Accreditation of Undergraduate Medical Training Programs: Practices in Nine Developing Countries as Compared with the United States. Education for Health. 2006;19(2):207–22. 16831802 10.1080/13576280600783570

[33] Frehywot S, Mullan F, Payne PW, Ross H. Compulsory service programmes for recruiting health workers in remote and rural areas: do they work? Bull World Health Organ. 2010;88(5):364–70. 20461136 10.2471/BLT.09.071605 PMC2865657

[34] WHO. The state of the health workforce in the WHO Africa region. Brazzaville, Congo: World Health Organisation; 2021.

[35] Alexander R, Badenhorst E, Gibbs T. Intervention programme: a supported learning programme for educationally disadvantaged students. Med Teach. 2005;27(1):66–70. 16147773 10.1080/01421590400016472

[36] Seggie J. MB ChB curriculum modernisation in South Africa – growing doctors for Africa. Afr J Health Prof Educ. 2010;2(1):8–14.

[37] Burch VC, Sikakana CN, Gunston GD, Shamley DR, Murdoch-Eaton D. Generic learning skills in academically-at-risk medical students: a development programme bridges the gap. Med Teach. 2013;35(8):671–7. 23782051 10.3109/0142159X.2013.801551

[38] Katz AA, Futter M, Mayosi BM. The intercalated BSc (Med) Honours/MB ChB and integrated MB ChB/PhD tracks at the University of Cape Town: models for a national medical student research training programme. S Afr Med J. 2014;104(2):111–3. 24893538 10.7196/samj.7639

[39] Eichbaum Q, Reid S, Coly A, Naidu T, Omaswa F. Conceptualizing Medical Humanities Programs in Low-Resource Settings in Africa. Acad Med. 2019;94(8):1108–14. 31094728 10.1097/ACM.0000000000002789

[40] Tsampiras C, Mkhwanazi N, Hume V. Inclusion, access and social justice: the rhizomic evolution of a field across a continent. Med Humanit. 2018;44(4):218–20. 30482813 10.1136/medhum-2018-011613

[41] Harden RM. What is a spiral curriculum? Med Teach. 1999;21(2):141–3. 21275727 10.1080/01421599979752

[42] Pattinson SR, McInerney P. Perceptions of changes made to a clinical skills curriculum in a medical programme in South Africa: A mixed methods study. Afr J Health Prof Educ. 2020;12(1):12–6.

[43] Botha GC, Crafford L. From understanding to action: a juncture-factor framework for advancing social responsiveness in health professions education. Front Med (Lausanne). 2024;11:1435472. 39712179 10.3389/fmed.2024.1435472 PMC11658996

[44] Rohwer A, Willems B, Young T. Taking stock of evidence-based healthcare in the undergraduate medical curriculum at Stellenbosch University: Combining a review of curriculum documents and input from recent graduates. Afr J Health Prof Educ. 2016;7(1):98–104.

[45] Mukinda FK, Goliath CD, Willems B, Zunza M, Dudley LD. Equipping medical graduates to address health systems challenges in South Africa: An expressed need for curriculum change. Afr J Health Prof Educ. 2016;7(1):86–91.

[46] Irlam J, Datay MI, Reid S, Alperstein M, Hartman N, Namane M, et al. How well do we teach the primary healthcare approach? A case study of health sciences course documents, educators and students at the University of Cape Town Faculty of Health Sciences. Afr J Health Prof Educ. 2021;13(1):83–92.

[47] Mnguni L. The Curriculum Ideologies Underlying the AfriMEDS Curriculum Framework for Undergraduate Medical and Dental Education in South Africa. International Medical Education. 2024;3(1):44–61.

[48] Matthews M, Van Wyk J. Speaking the language of the patient: indigenous language policy and practice. South African Family Practice. 2015;58(1):30–1.

[49] Mohamed Z, Roche S, Claassen J, Jama Z. Students’ perceptions of the effectiveness of additional language tuition in the University of Cape Town MBChB programme: A descriptive cross-sectional study. Afr J Prim Health Care Fam Med. 2019;11(1):e1–10. 10.4102/phcfm.v11i1.2121 PMC6852487 31714121

[50] Matthews MG, Van Wyk JM. Improving communication in the South African healthcare context. Afr J Health Prof Educ. 2018;10(4):194–8.

[51] Mokhachane M, Wyatt T, Kuper A, Green-Thompson L, George A. Graduates' Reflections on Professionalism and Identity: Intersections of Race, Gender, and Activism. Teach Learn Med. 2023:1–11. 10.1080/10401334.2023.2224306 37334670

[52] Mokhachane M, George A, Wyatt T, Kuper A, Green-Thompson L. Rethinking professional identity formation amidst protests and social upheaval: a journey in Africa. Adv Health Sci Educ Theory Pract. 2023;28(2):427–52. 36301374 10.1007/s10459-022-10164-0 PMC10169886

[53] Mokhachane M, Green-Thompson L, George A, Wyatt T, Kuper A. Medical students’ views on what professionalism means: an Ubuntu perspective. Adv Health Sci Educ Theory Pract. 2024;29(3):841–57. 37710029 10.1007/s10459-023-10280-5 PMC11208190

[54] SAQA. Core Competencies for Undergraduate Students in the Clinical Associate, Dentistry and Medical Teaching and Learning programmes in South Africa. . Pretoria, South Africa: South African Qualifications Authority (SAQA); 2012.

[55] Irlam J, Keikelame M, Vivian L. Integrating the primary health care approach into a medical curriculum: a programme logic model. Afr J Health Prof Educ. 2009;1(1):8–11.

[56] Sukrajh V, Adefolalu AO, Louw AJN. Promoting active learning in medical education using the peer teaching model: perceptions of senior medical students. SN Social Sciences. 2021;1(7):158(1-17).

[57] van Staden D. Investing in health professions education: A national development imperative for South Africa. S Afr J Higher Educ. 2021;35(1):231–45.

[58] Dreyer AR, Rispel LC, Cheng M. Context, types, and utilisation of decentralised training platforms in undergraduate medical education at four South African universities: Implications for universal health coverage. Cogent Education. 2021;8(1):1–16.

[59] de Villiers M, van Schalkwyk S, Blitz J, Couper I, Moodley K, Talib Z, et al. Decentralised training for medical students: a scoping review. BMC Med Educ. 2017;17(1):196. 29121923 10.1186/s12909-017-1050-9 PMC5680751

[60] Muller J. The collaborative care project: A practice-based approach to interprofessional education in a primary healthcare setting in South Africa. Educ Health (Abingdon). 2019;32(3):141–5. 32317423 10.4103/efh.EfH_276_19

[61] Kitema GF, Laidlaw A, O’Carroll V, Sagahutu JB, Blaikie A. The status and outcomes of interprofessional health education in sub-Saharan Africa: A systematic review. J Interprof Care. 2024;38(1):133–55. 36739570 10.1080/13561820.2023.2168631

[62] Muller J, Reardon C, Coetzee F, Bester J, Dube K, Hanekom S, et al. Transformative learning through participation: experiences at a rural clinical training site in South Africa. BMC Med Educ. 2022;22(1):183. 35296325 10.1186/s12909-022-03233-w PMC8928645

[63] Mlambo M, Dreyer A, Dube R, Mapukata N, Couper I, Cooke R. Transformation of medical education through Decentralised Training Platforms: a scoping review. Rural Remote Health. 2018;18(1):4337. 29522688 10.22605/RRH4337

[64] Jacobs C, Van Schalkwyk S, Blitz J, Volschenk M. Advancing a social justice agenda in health professions education. Crit Stud Teach Learn. 2020;8(2):112–31.

[65] Muller J, Meyer R, Bantjes J, Archer E, Couper I. Handle with Care: Transformative Learning as Pedagogy in an Under-Resourced Health Care Context. Teach Learn Med. 2024:1–10. 10.1080/10401334.2024.2332885 38634761

[66] Mapukata N, Masinire A, Nkambule T. Parallels and Divergences in Decentralised Training Approaches: Reflecting on the Net Value of Implementing a Collaborative Model in a South African University. In: Ndofirepi AP, Masinire A, editors. Rurality, Social Justice and Education in Sub-Saharan Africa Volume II: Theory and Practice in Higher Education. Cham: Springer International Publishing; 2020. p. 127–45.

[67] Brits H, Bezuidenhout J, Van der Merwe LJ, Joubert G. Assessment practices in undergraduate clinical medicine training: What do we do and how we can improve? Afr J Prim Health Care Fam Med. 2020;12(1):e1–7. 10.4102/phcfm.v12i1.2341 PMC7433310 32634019

[68] Walubo A, Burch V, Parmar P, Raidoo D, Cassimjee M, Onia R, et al. A model for selecting assessment methods for evaluating medical students in African medical schools. Acad Med. 2003;78(9):899–906. 14507620 10.1097/00001888-200309000-00011

[69] Burch V. Medical Education in South Africa: assessment practices in a developing country. 2007.

[70] Burch VC, Seggie JL. Use of a structured interview to assess portfolio-based learning. Med Educ. 2008;42(9):894–900. 18715487 10.1111/j.1365-2923.2008.03128.x

[71] Mennin S, Burch V, Kwizera E, Troncon L, Singh T, Sood R. Culture, Medical Education and Assessment. International Best Practices for Evaluation in Health Professions. London: CRC Press; 2022. p. 229–55.

[72] Sims D, Lucio-Ramirez CA, Cilliers FJ. Factors influencing clinician-educators’ assessment practice in varied Southern contexts: a health behaviour theory perspective. Advances in Health Sciences Education. 2024. 10.1007/s10459-024-10341-3 PMC11926032 38811446

[73] Hanekom SD, Unger M, Cilliers F. Deriving criteria by which to determine core curriculum content: A high engagement process. Afr J Health Prof Educ. 2014;6(2):180–4. 10.7196/AJHPE.496.

[74] Dudley L, Young T, Rohwer A, Willems B, Dramowski A, Goliath C, et al. Fit for purpose? A review of a medical curriculum and its contribution to strengthening health systems in South Africa African Journal of Health Professions Education. 2015;7(1):81–5.

[75] Pattinson SR, Savelberg H, Atherley A. Not ready in the ways that count– a qualitative exploration of junior doctor’s perceived preparedness for practice using Legitimation Code Theory. Adv Health Sci Educ Theory Pract. 2024. 10.1007/s10459-024-10380-w. 10.1007/s10459-024-10380-w PMC12119643 39373869

[76] Botha GC, Adefolalu AO. Curriculum Mapping of Undergraduate Medical Programmes: Recommendations for Future Directions. European Journal of Education and Pedagogy. 2021;2(6):1–7.

[77] Ingratta AM, Mabizela SE, George AZ, Green-Thompson L. Undergraduate medical students’ readiness for online learning at a South African university: Implications for decentralised training. Afr J Health Prof Educ. 2022;14(2):66–71.

[78] Jacob N, Cilliers F, Begg K, Green-Thompson L. Navigating COVID-19: Preparing medical students in a time of pandemic. Afr J Health Prof Educ. 2021;13(1):10–1.

[79] Schmutz AMS, Jenkins LS, Coetzee F, Conradie H, Irlam J, Joubert EM, et al. Re-imagining health professions education in the coronavirus disease 2019 era: Perspectives from South Africa. Afr J Prim Health Care Fam Med. 2021;13(1):e1–5. 10.4102/phcfm.v13i1.2948 PMC8424738 34476976

[80] Noorbhai H, Ojo TA. mHealth and e-Learning in health sciences curricula: a South African study of health sciences staff perspectives on utilisation, constraints and future possibilities. BMC Med Educ. 2023;23(1):189. 36978117 10.1186/s12909-023-04132-4 PMC10043831

[81] Noorbhai H, Sims D, Hartman N. South African health sciences students’ perspectives on utilisation, constraints and future possibilities of mHealth and e-Learning. Discov Educ. 2023;2(1):24. 10.1186/s12909-023-04132-4 PMC10043831 36978117

[82] Enoch LC, Abraham RM, Singaram VS. A comparative analysis of the impact of online, blended, and face-to-face learning on medical students’ clinical competency in the affective, cognitive, and psychomotor domains. BMC Med Educ. 2022;22(1):753. 36320031 10.1186/s12909-022-03777-x PMC9628081

[83] Titus S. Implementing extended reality (XR) and artificial intelligence (AI) in health professions education in southern Africa. Afr J Health Prof Educ. 2024;16(2):685–94.

[84] Naidoo S, Bottomley D, Naidoo M, Donnelly D, Thaldar DW. Artificial intelligence in healthcare: Proposals for policy development in South Africa. S Afr J Bioeth Law. 2022;15(1):11–6. 36061984 10.7196/sajbl.2022.v15i1.797 PMC9439582

[85] Bedoll D, van Zanten M, McKinley D. Global trends in medical education accreditation. Hum Resour Health. 2021;19(1):70. 34016122 10.1186/s12960-021-00588-x PMC8136216

[86] Rashid MA, Ali SM, Dharanipragada K. Decolonising medical education regulation: a global view. BMJ Glob Health. 2023;8(6):e011622. 10.1136/bmjgh-2022-011622 PMC10277086 37311579

[87] Karunathilake IM, Grant J, Rashid MA, Samarasekara D, Prihatiningsih ST, de Abrew A, et al. The Need for Accreditation in Medical Education to be based on Regional and National Health Priorities. South-East Asian J Med Educ. 2022;16(2):1–6.

[88] Sims D. When I say ... global south and global north. Med Educ. 2023;58(3):286–7. 10.1111/medu.15263. 10.1111/medu.15263 37963543

[89] Kiguli-Malwadde E, Talib ZM, Wohltjen H, Connors SC, Gandari J, Banda SS, et al. Medical education departments: a study of four medical schools in Sub-Saharan Africa. BMC Med Educ. 2015;15:109. 26126821 10.1186/s12909-015-0398-y PMC4486688

[90] ASSAF. Reconceptualising Health Professions Education in South Africa: Consensus Study Report. Pretoria, South Africa; 2018 March 2018.

[91] van Schalkwyk S, O’Brien BC, van der Vleuten C, Wilkinson TJ, Meyer I, Schmutz AMS, et al. Exploring perspectives on health professions education scholarship units from sub-Saharan Africa. Perspect Med Educ. 2020;9(6):359–66. 32930985 10.1007/s40037-020-00619-8 PMC7718360

[92] Van Schalkwyk S, Kiguli-Malwadde E, Budak JZ, Reid MJA, de Villiers MR. Identifying research priorities for health professions education research in sub-Saharan Africa using a modified Delphi method. BMC Med Educ. 2020;20(1):443. 33208149 10.1186/s12909-020-02367-z PMC7672834

[93] Greysen SR, Dovlo D, Olapade-Olaopa EO, Jacobs M, Sewankambo N, Mullan F. Medical education in sub-Saharan Africa: a literature review. Med Educ. 2011;45(10):973–86. 21916938 10.1111/j.1365-2923.2011.04039.x

[94] Mokhachane M, Green-Thompson L, Wyatt TR. Voices of Silence: Experiences in Disseminating Scholarship as a Global South Researcher. Teach Learn Med. 2023;36(2):1–9. 10.1080/10401334.2023.2181815 36843331

[95] Naidu T, Cartmill C, Swanepoel S, Whitehead C. Shapeshifters: Global South scholars and their tensions in border-crossing to Global North journals. BMJ Glob Health. 2024;9:1–10. 10.1136/bmjgh-2023-014420 PMC11029397 38724078

[96] Naidu T. The personal is political in the struggle for equity in global medical education research and scholarship. Med Teach. 2023;45(9):991–6. 37200518 10.1080/0142159X.2023.2206535

[97] Kusurkar RA, Lilley P, Harden R. Medical Teacher's equity diversity inclusion policy. Med Teach. 2024;46(6):1–2. 10.1080/0142159X.2024.2334394 38557241

[98] Wyatt TR, Bullock JL, Andon A, Odukoya EJ, Torres CG, Gingell G, et al. Editors as Gatekeepers: One Medical Education Journal’s Efforts to Resist Racism in Scholarly Publishing. Acad Med. 2023;98(12):1406–12. 37378634 10.1097/ACM.0000000000005303

[99] Dimassi Z, Ibrahim H. Representation in Health Professions Education: Striving for an Inclusive Health Professions Education Community. Perspect Med Educ. 2023;12(1):438–43. 37901883 10.5334/pme.883 PMC10607562

[100] Manan MR, Nawaz I, Rahman S, Manan H. Diversity, equity, and inclusion in medical education journals: An evaluation of editorial board composition. Med Teach. 2023;46(2):1–9. 10.1080/0142159X.2023.2249212 37634062

[101] Yip SWL, Rashid MA. Editorial diversity in medical education journals. Clin Teach. 2021;18(5):523–8. 34047056 10.1111/tct.13386

[102] Sommerville T, Singaram VS. Exploring demographic influences on students’ academic performance over a five-year programme. S Afr J High Educ. 2018;32(2):273–87.

[103] George A, Blaauw D, Thompson J, Green-Thompson L. Doctor retention and distribution in post-apartheid South Africa: tracking medical graduates (2007–2011) from one university. Hum Resour Health. 2019;17(1):100. 31842879 10.1186/s12960-019-0439-4 PMC6916458

[104] Sims DA, Naidu T. How to ... do decolonial research. Clin Teach. 2024;21(6):e13806. 39293474 10.1111/tct.13806

[105] Singaram VS, Sofika DAN. “Growing as a Stronger Clinician in Adverse Conditions”—A Snapshot of Clinical Training during COVID-19. Educ Sci. 2022;12(3):156.

